# Impact of circulating lymphoma cells at diagnosis on outcomes in patients with Waldenstrom macroglobulinemia

**DOI:** 10.3389/fonc.2023.1264387

**Published:** 2023-09-13

**Authors:** Ansel Nalin, Qiuhong Zhao, Timothy Voorhees, David Bond, Yazeed Sawalha, Walter Hanel, Audrey Sigmund, Kaitlin Annunzio, Lapo Alinari, Robert Baiocchi, Kami Maddocks, Dan Jones, Beth Christian, Narendranath Epperla

**Affiliations:** ^1^ Department of Medicine, The Ohio State University, Columbus, OH, United States; ^2^ Division of Hematology, The James Cancer Hospital and Solove Research Institute, The Ohio State University, Columbus, OH, United States; ^3^ Department of Pathology, The Ohio State University, Columbus, OH, United States

**Keywords:** circulating lymphoma, CL, Waldenström macroglobulinemia, WM, progression-free survival

## Abstract

Given the paucity of data surrounding the prognostic relevance of circulating lymphoma (CL) in Waldenström macroglobulinemia (WM), we sought to evaluate the impact of CL at diagnosis on outcomes in patients with WM. Patients were divided into CL+ and CL- based on the results of flow cytometry. The endpoints included assessing progression-free survival (PFS), overall survival (OS), and diagnosis-to-treatment interval (DTI) between the two groups. Among the 308 patients with WM, 69 met the eligibility criteria with 42 and 27 in CL+ and CL- groups, respectively. The two groups were well balanced in regard to all the baseline characteristics. The ORR was numerically higher in the CL+ group compared to the CL-group (81% versus 61%, respectively), however, the CR+VGPR rates were similar between the two groups. The median PFS was not significantly different between the two groups (6.3 years in the CL- group versus not reached [NR] in the CL+ group) regardless of the first-line therapy. There was no significant difference in median OS between the CL- and CL+ groups (13 years versus NR). Although the median DTI was shorter in the CL+ group compared to CL- group, the significance was lost in the multivariable analysis. In this study (largest-to-date) evaluating the impact of CL on outcomes in patients with newly diagnosed WM, we did not find the prognostic utility of CL in WM. Future studies should explore the correlation of CL with other biological factors that impact the outcomes in WM patients.

## Introduction

Waldenström macroglobulinemia (WM) is the most common subtype of the generally indolent lymphoplasmacytic lymphoma (LPL) type of B-cell non-Hodgkin lymphoma (NHL) (1). It is characterized by infiltration of lymphoplasmacytic cells in the bone marrow and/or spleen and lymph nodes along with excess IgM monoclonal protein in the peripheral blood (PB) (2). Mutations of *MYD88* and *CXCR4* genes are commonly implicated in WM, and ongoing research seeks to identify other genetic abnormalities involved in its pathogenesis (3).

Patients with WM present with symptoms related to the infiltration of malignant cells in specific tissues and or excess IgM protein in the blood. WM is diagnosed based on evidence of lymphoplasmacytic infiltration in the bone marrow or lymph nodes, presence of an IgM spike on serum protein electrophoresis, and an abnormal ratio of kappa and lambda chains on serum free light chain assay (2). A subset of patients with WM present with circulating lymphoma (CL) cells in the PB at the time of diagnosis (4–6). However, the prognostic relevance of CL in WM is unknown. Hence, we sought to evaluate the impact of CL in patients with newly diagnosed WM.

## Methods

### Study design

This is a single institution retrospective cohort study of patients diagnosed with WM in or after 2010. The study was approved by the institutional review board and was conducted in compliance with the Declaration of Helsinki. To be eligible, patients must have received treatment for WM and had PB immunophenotyping via flow cytometry performed at diagnosis. CL was defined as detectable clonally restricted B-cells that matched the actual or expected CD5-CD10- B-cell immunophenotype of LPL. Any ambiguous cases that did not fit the diagnosis of the WM variant of LPL (e.g., non-IgM paraprotein and absence of bone marrow involvement) were excluded.

Demographic and clinical patient data were obtained from the electronic medical record. Laboratory values were collected for variables known to be associated with WM prognosis, including hemoglobin, platelet count, β2-microglobulin, monoclonal IgM, albumin, and LDH (7, 8). The International Prognostic Scoring System for WM (IPSSWM) was used to assign a prognostic score for each patient (7). Treatment response was assessed via The International Working Group on Waldenström Macroglobulinemia criteria (9).

### Study objectives and definitions

Patients who met the study inclusion criteria were classified based on the presence or absence of CL at the time of diagnosis into two groups: those with positive PB flow cytometry (CL+) and those with negative PB flow cytometry (CL-). The primary endpoint of this study was to evaluate progression-free survival (PFS) between the two groups. Secondary endpoints included evaluation of overall survival (OS) and diagnosis to treatment interval (DTI).

PFS was defined as the time from the start of first-line therapy until lymphoma relapse/progression or death from any cause, censoring from the last clinical assessment time. OS was defined as the time from the start of first-line therapy until death from any cause or censoring at the last clinical assessment. DTI was defined as the time from diagnosis to initiation of first-line systemic therapy Skeletal involvement was defined as any of these findings including osteopenia, marrow space widening, endosteal erosions, osteolytic lesions, etc. (10–12) Complex karyotype was defined as the presence of at least three chromosomal aberrations in at least two cells.

### Statistical analysis

Demographic and disease characteristics were summarized using median and range for continuous variables, and frequency and percentage for categorical variables, compared among study groups using the Wilcoxon signed rank test, and Chi-square test, or Fisher’s exact test, respectively. PFS was estimated using the Kaplan-Meier method and compared between two groups using the log-rank test. Cox proportional hazard regression models were used to estimate the hazard ratios for risk of progression or death. The multivariable Cox model was built including all the variables associated with PFS in the univariable model with p<0.10. The proportional hazard assumption was checked using Schoenfeld residuals after fitting Cox models. OS was compared using the log-rank test. Analyses were performed using Stata version 17 (StataCorp, College Station, TX), and all statistical tests were two-sided with a type 1 error of <0.05 indicating statistical significance. All estimates were reported with 95% confidence intervals (95% CI).

## Results

### Baseline characteristics

Among the 308 patients with WM, 87 had flow performed at diagnosis and after excluding patients who did not meet the eligibility criteria, 69 cases remained that were included in the final analysis (see Consort diagram, **Figure S1**). Among these 42 (61%) had detectable CL at diagnosis. The median age at diagnosis for all included patients was 63 years. There was male predomiance and most of the patients were white and had good performance status (ECOG performance status of 0-1). There were no statistically significant differences in IgM level, presence of B symptoms, skeletal or bone marrow involvement, *MYD88* or *CXCR4* mutations, or IPSSWM prognostic scores between the CL+ and CL- groups. 64% of the patients received immunochemotherapy as first-line therapy relative to 22% who received rituximab monotherapy. This was not significantly different between the CL- and CL+ groups. The most commonly administered immunochemotherapy was BR (bendamustine and rituximab) in the current study (n=32), which was numerically higher in the CL+ group (n=22, 52%) compared to CL- group (n=10, 37%). The median follow-up was 4.27 years. **Table 1** shows the baseline characteristics of the patient population according to the presence or absence of CL.

**Table 1:** Baseline Characteristics.

Variable	All patients N=69 (%)	CL- N=27 (%)	CL+ N=42 (%)	P-value
Median age, yrs (range)	63 (40-94)	63 (40-78)	63.5 (45-94)	0.61
Sex				0.93
Male	43 (62)	17 (63)	26 (62)	
Female	26 (38)	10 (37)	16 (38)	
Race				0.73
White	59 (86)	24 (89)	35 (83)	
Black	10 (15)	3 (11)	7 (17)	
ECOG PS				0.99
0-1	64 (93)	25 (93)	39 (93)	
≥ 2	5 (7)	2 (7)	3 (7)	
IgM level				0.58
≤ 1000	19 (28)	9 (33)	10 (24)	
1001 – 3000	25 (36)	10 (37)	15 (36)	
> 3000	25 (36)	8 (30)	17 (41)	
B Symptoms	7 (10)	2 (7)	5 (12)	0.70
Skeletal Involvement	4 (6)	2 (7)	2 (5)	0.64
Bone Marrow Involvement	66 (96)	24 (89)	42 (100)	0.06
MYD88 mutation				0.28
No	14 (28)	7 (37)	7 (23)	
Yes	36 (72)	12 (63)	24 (77)	
Missing	19	8	11	
CXCR4 mutation				0.68
No	20	9	11	
Yes	7 (26)	4 (31)	3 (21)	
Missing	42	14	28	
Low Serum Albumin	19 (28)	7 (27)	12 (29)	0.88
LDH > ULN	18 (27)	4 (15)	14 (33)	0.16
B2M				0.23
Elevated	39 (75)	11 (65)	28 (80)	
Not elevated	13 (25)	6 (35)	7 (20)	
Missing	17	10	7	
Complex karyotype	27 (44)	9 (38)	18 (47)	0.45
Prognostic score (IPSSWM)				0.40
0-1	35 (51)	16 (59)	19 (45)	
2	18 (26)	7 (26)	11 (26)	
3-4	16 (23)	4 (15)	12 (29)	
First-line therapy				0.28
Rituximab monotherapy	15 (22)	8 (30)	7 (17)	
Immunochemotherapy	44 (64)	17 (63)	27 (64)	
Others	10 (14)	2 (7)	8 (19)	

Yrs, years; CL, circulating lymphoma; ECOG PS, Eastern Cooperative Oncology Group performance status; LDH, lactate dehydrogenase; ULN, upper limit of normal; B2M, beta-2-microglobulin; IPSSWM, International Prognostic Scoring System for Waldenstrom Macroglobulinemia.

### Hepatitis B and C viral status

There were no cases of positive hepatitis C viral antibody in our study. While there were no patients with active hepatitis B viral (HBV) infection based on the serological studies, there were 5 patients (2 in CL+ and 3 in CL-) with positive HBV core antibody but undetectable HBV DNA level (by PCR). All these patients were given antiviral prophylaxis (entecavir or tenofovir) during treatment and for at least 6 months following the last treatment and 12 months following the last rituximab infusion. None of the patients in our study had HBV reactivation.

### Response rates

The overall response rate (ORR) was 74% (n=51) with 42% (n=29) achieving a complete response or very good partial response and 32% (n=22) achieving a partial response. The ORR was numerically higher in CL+ group compared to CL- group (81% versus 61%, respectively), while the CR+VGPR rates were comparable between the two groups (40% versus 42%, respectively). The breakdown of the response rate between the CL- and CL+ groups is shown in **Table S1**.

### Progression-free survival

**Figure 1:**
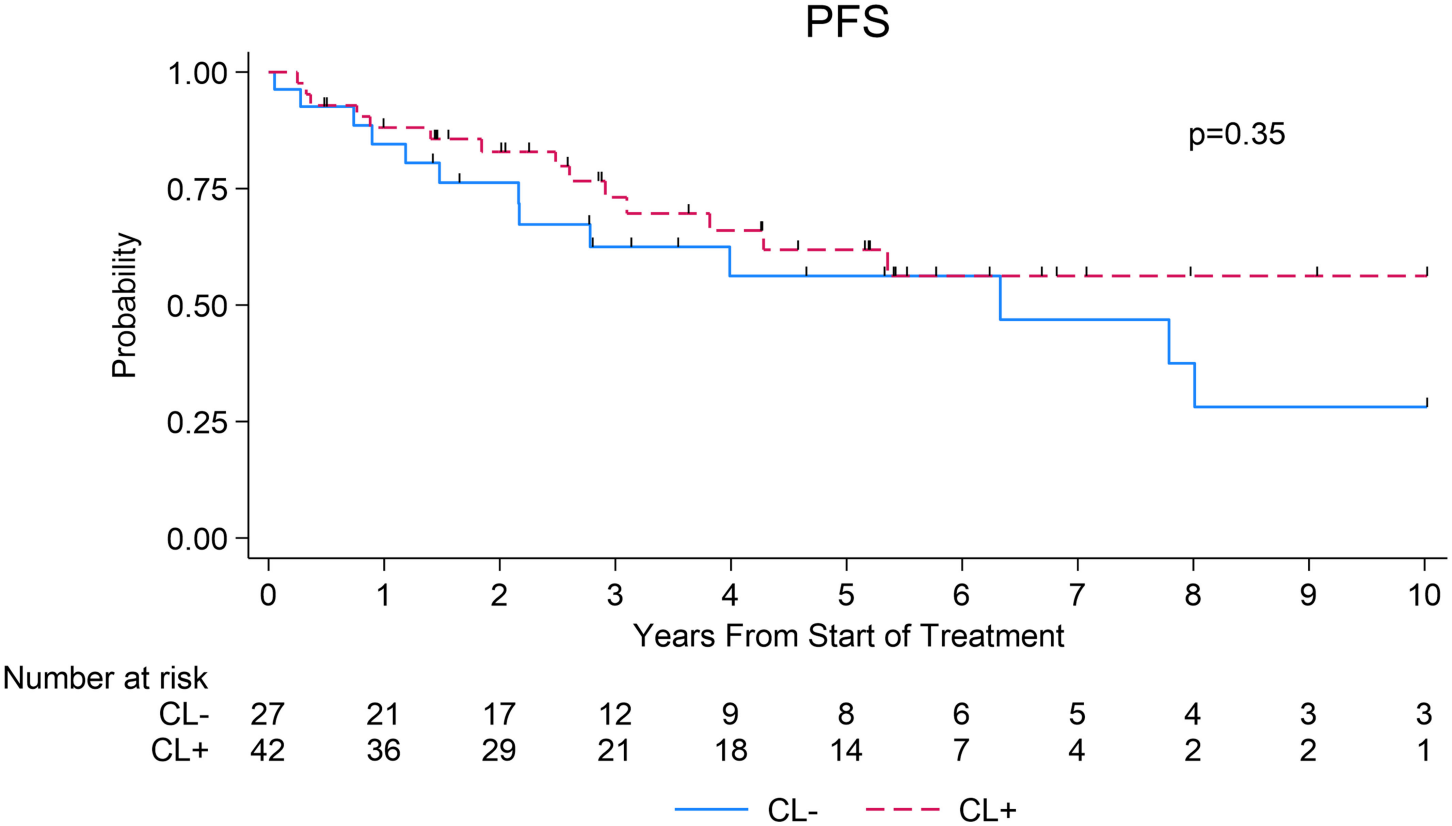
Progression-free survival after first-line systemic therapy among patients with WM, stratified by presence or absence of circulating lymphoma cells at diagnosis.

The median PFS was 6.3 years (95% CI=2.2 to not reached [NR]) in the CL- group compared to NR in the CL+ group (95% CI=3.8 to NR), which was not statistically significant (log-rank p=0.35; **Figure 1**). The 3- and 5-year PFS estimates in the CL+ group were 73% (95% CI=55-85%) and 62% (95% CI=43-76%) compared to 63% (95% CI=40-79%) and 56% (95% CI=33-74%) in the CL- group, respectively (log-rank p=0.35, **Figure 1**).

When evaluating the outcomes based on the type of first-line systemic therapy received, there was no significant difference in median PFS (in years) between CL+ and CL- groups among those who received rituximab monotherapy (NR [95% CI: 0.76 to NR] *vs* NR [95% CI=0.05 to NR]) (p=0.35, **Figure S2A**) or rituximab plus combination chemotherapy (R-chemotherapy) (NR [95% CI=3.10 to NR] *vs* 6.33 [95% CI=2.16-8.01]) (p=0.20, **Figure S2B**). In the univariate Cox model, factors associated with inferior PFS included age and IPSSWM prognostic score. After adjusting for these factors in a multivariable Cox model, presence of CL was not associated with significantly inferior PFS (HR=0.50, 95% CI=0.22-1.10, p=0.08, **Table 2**). Only IPSSWM (HR=4.42, 95% CI=1.35 – 14.42, p=0.01, **Table 2**) remained associated with significantly inferior PFS in the multivariable analysis.

**Table 2:** Univariate and Multivariable Cox modeling on progression-free survival analysis among patients who received systemic therapy.

Variable	Univariate		Multivariable	
	HR (95% CI)	P-value	HR (95% CI)	P-value
Circulating Lymphoma				
CL-	1.00		1.00	
CL+	0.69 (0.32 – 1.49)	0.35	0.50 (0.22 – 1.10)	0.08
Age at dx (yrs)	1.04 (1.00 – 1.08)	0.04	1.02 (0.98 – 1.06)	0.38
Sex				
Male	1.00		–	–
Female	0.90 (0.42 – 1.94)	0.79	–	–
Race				
White	1.00		–	–
Black	0.30 (0.07 – 1.30)	0.11	–	–
ECOG PS				
0-1	1.00		–	–
≥ 2	1.90 (0.56 – 6.40)	0.30	–	–
IgM level				
≤ 1000	1.00			
1001 – 3000	0.50 (0.18 – 1.33)	0.16	–	–
> 3000	0.89 (0.37 – 2.15)	0.79	–	–
B Symptoms				
No	1.00		–	–
Yes	0.34 (0.05 – 2.51)	0.29	–	–
Albumin Low				
No	1.00		–	–
Yes	1.03 (0.45 – 2.35)	0.95	–	–
LDH > ULN				
No	1.00		–	–
Yes	1.54 (0.69 – 3.47)	0.30	–	–
Elevated B2M				
No	1.00		–	–
Yes	2.22 (0.65 – 7.60)	0.20	–	–
Complex Karyotype				
No	1.00		–	–
Yes	0.98 (0.44 – 2.17)	0.96	–	–
Prognostic score (IPSSWM)				
0-1	1.00			
2	2.96 (1.12 – 7.83)	**0.03**	2.70 (0.90 – 8.12)	0.08
3-4	4.65 (1.70 – 12.68)	**0.003**	4.42 (1.35 – 14.42)	**0.01**
First-line Treatment				
Rituximab monotherapy	1.00			
Immunochemotherapy	1.56 (0.52 – 4.70)	0.42	1.12 (0.35 – 3.61)	0.85
Others	3.05 (0.84 – 11.13)	0.09	1.54 (0.36 – 6.54)	0.56

Yrs, years; CL, circulating lymphoma; ECOG PS, Eastern Cooperative Oncology Group performance status; LDH, lactate dehydrogenase; ULN, upper limit of normal; B2M, beta-2-microglobulin; IPSSWM, International Prognostic Scoring System for Waldenstrom Macroglobulinemia.

### Diagnosis to treatment interval

Patients in the CL- group had a significantly longer median DTI (4.1 months; 95% CI=0.84-21.6) compared to the CL+ group (1.1 months; 95% CI=0.06-0.18) (p=0.048; **Figure S3**). The percentage of patients who started treatment at 1 year in the CL+ versus CL- groups was 83% *vs* 63%, respectively. CL was associated with a shorter DTI in the univariate analysis, however, after adjusting for other factors in the multivariable analysis this association was no longer significant (**Table S2**).

### Overall survival

**Figure 2:**
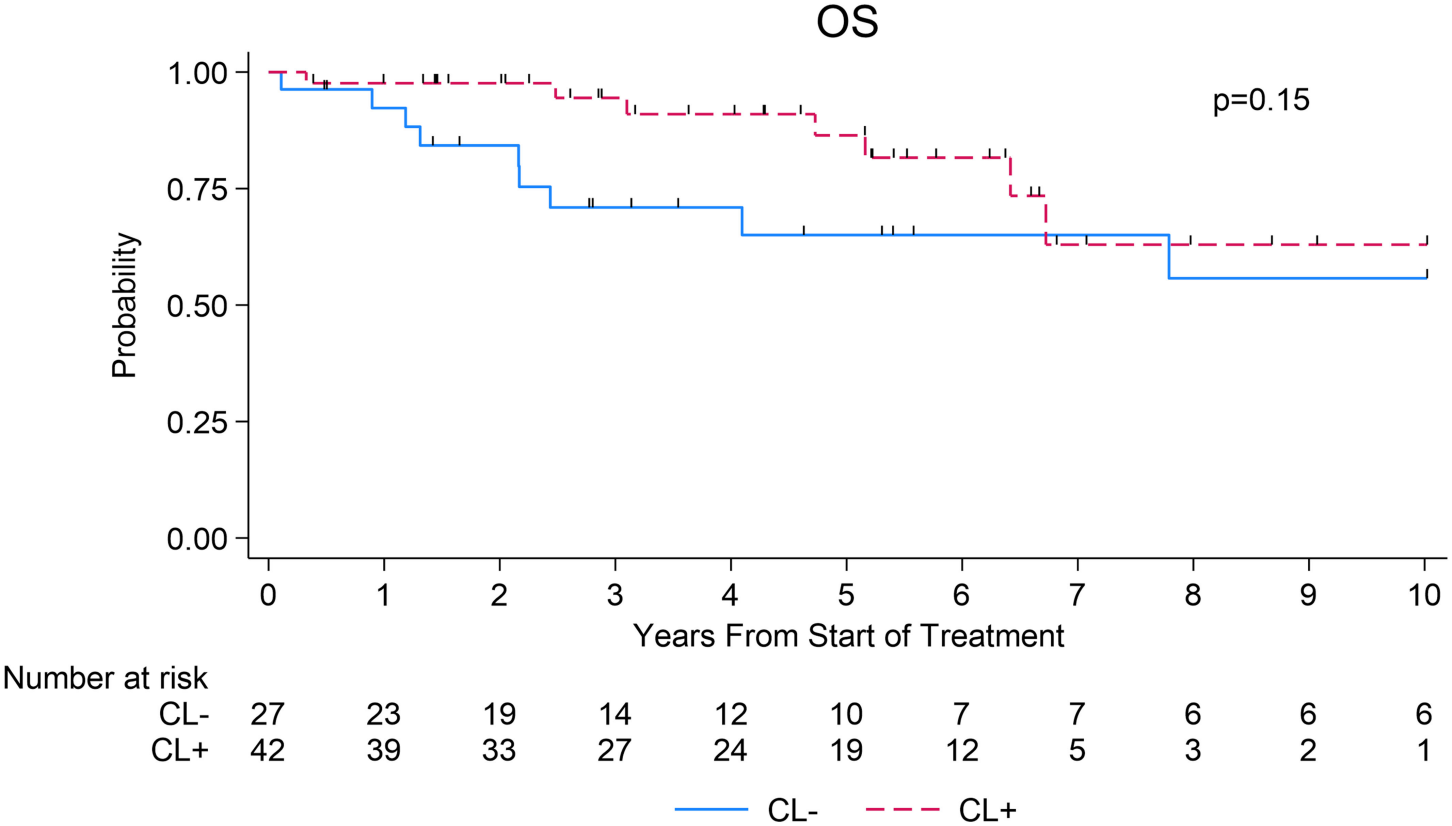
Overall survival after first-line systemic therapy among patients with WM.

There was no significant difference in median OS between the CL- group (13.0 years; 95% CI=2.4 to NR) and CL+ group (NR; 95% CI: 6.4 to NR), (p =0.15; **Figure 2**). The 3- and 5-year OS estimates in the CL+ group were 94% (95% CI=79-99%) and 86% (95% CI=67-95%) compared to 71% (95% CI=48-85%) and 65% (95% CI=41-81%) in the CL- group, respectively (log-rank p=0.15, **Figure 2**).

## Discussion

In this retrospective cohort study, we evaluated the impact of CL at the time of diagnosis in patients with WM and made several important observations. First, the presence of CL at diagnosis did not have a significant impact on PFS in WM regardless of the first-line therapy. Second, there was no significant difference in median OS between the CL- and CL+ groups. Third, although the median DTI was shorter in the CL+ group compared to CL- group, there was no significant difference between the two groups in regard to the DTI after adjusting for the significant covariates in the multivariable analysis. To our knowledge, this is the first in-depth study evaluating the effect of CL (or lack thereof) at diagnosis on outcomes in patients with WM.

CL was previously reported in patients with WM (4–6) (**Table S3**); however, these studies were limited by a small sample size precluding detailed analysis. In one study, nine of sixteen patients with WM had increased PB monoclonal lymphocytes. In that study patients with CL presented with acute clinical symptoms including lymphadenopathy and splenomegaly (4) and the authors hypothesized that disease progression was a major factor leading to CL, however, this study did not evaluate whether CL was associated with differences in survival outcomes. In our study, we found no significant difference in the survival (PFS or OS) between CL+ and CL- groups. These findings are similar to the recently reported outcomes in patients with MZL presenting with CL (13).

DTI was previously shown to be an important prognostic factor in patients with newly diagnosed diffuse large B-cell lymphoma (14) and mantle cell lymphoma (15) and was associated with adverse clinical factors. In line with the previously reported studies, shorter DTI was associated with adverse clinical factors (high IgM level [>3000], presence of B symptoms, low albumin, presence of complex karyotype, and intermediate and high IPSSWM) in patients with newly diagnosed WM in our study. However, in contrast to those earlier studies (14, 15), DTI did not have a prognostic utility (in the multivariable analysis) in patients with newly diagnosed WM.

The study is subjected to the inherent limitations of a retrospective study design including non-uniform treatment selection, short follow-up (median follow-up was <5 years), and performance of PB flow cytometry at diagnosis. We did not collect information on patients who did not have flow cytometry at diagnosis precluding our ability to learn the differences between the patients who had flow checked versus not. Due to the limited sample size, we could not study the impact of genomic markers on the outcomes in patients with or without CL.

In conclusion, this is the largest study to date to evaluate the impact of CL on outcomes in patients with newly diagnosed WM. In this study, we found that the presence of CL at diagnosis in WM was not associated with inferior PFS or OS. Future studies should explore the correlation of CL with other biological factors that impact the outcomes in patients with WM.

## Data availability statement

The raw data supporting the conclusions of this article will be made available by the authors, without undue reservation.

## Ethics statement

The studies involving humans were approved by The Ohio State University and James Cancer Center. The studies were conducted in accordance with the local legislation and institutional requirements. The ethics committee/institutional review board waived the requirement of written informed consent for participation from the participants or the participants’ legal guardians/next of kin because This is a retrospective cohort study.

## Author contributions

AN: Data curation, Writing – original draft. QZ: Formal Analysis, Writing – review & editing. TV: Writing – review & editing. DB: Writing – review & editing. YS: Writing – review & editing. WH: Writing – review & editing. AS: Writing – review & editing. KA: Writing – review & editing. LA: Writing – review & editing. RB: Writing – review & editing. KM: Writing – review & editing. DJ: Writing – review & editing. BC: Writing – review & editing. NE: Conceptualization, Data curation, Investigation, Methodology, Supervision, Writing – original draft, Writing – review & editing.
