# Supplementary material for: Impact of circulating lymphoma cells at diagnosis on outcomes in patients with Waldenstrom macroglobulinemia
Source: Front Oncol. 2023 Sep 13;13:1264387. doi: 10.3389/fonc.2023.1264387 (PMC10533994; doi:10.3389/fonc.2023.1264387)
Supplement: Supplementary file 1 [file DataSheet_1.docx]

**SUPPLEMENTAL APPENDIX**

**Table of Contents** 1

**Study Design:** Figure S1 2

**PFS among CL- and CL+ groups, stratified by type of first-line therapy:** Figure S2 3

**Time from diagnosis to initiation of systemic therapy between CL- and CL+ groups:** Figure S3 4

**Response rates to first-line therapy between CL- and CL+ groups:** Table S1 5

**Cox modeling on time to treatment among patients who received systemic therapy:** Table S2 6

**Studies that reported the incidence of circulating lymphoma cells in WM:** Table S3 7

**Figure S1. Consort Diagram.**

**
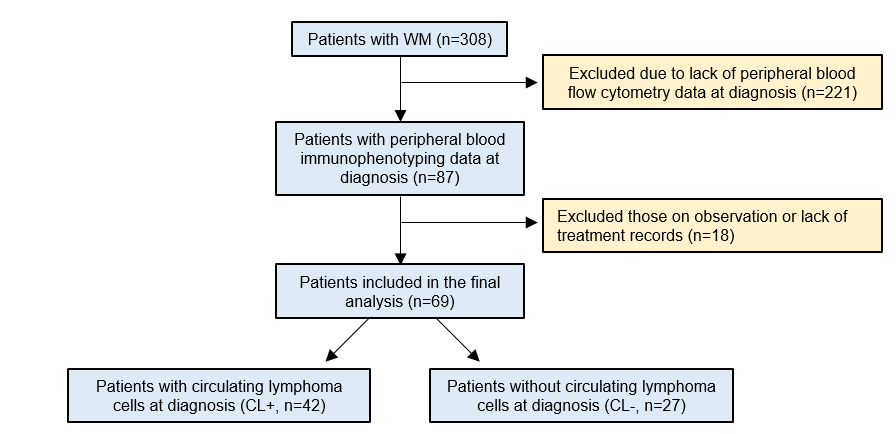
**

**Figure S2.** **Progression-free survival among CL- and CL+ groups, stratified by type of first-line therapy.**

**Figure S3. Time from diagnosis to initiation of first-line systemic therapy among patients with WM.**

**Table S1. Response rates between CL+ and CL- groups in patients with WM**

| **Variable** | **All patients**  **N=69 (%)** | **CL-**  **N=27 (%)** | **CL+**  **N=42 (%)** | **P-value** |
| --- | --- | --- | --- | --- |
| Response to treatment |  |  |  | 0.08 |
| CR+VGPR | 29 (42) | 12 (42) | 17 (40) |  |
| Partial response | 22 (32) | 5 (19) | 17 (41) |  |
| Minor response | 4 (6) | 1 (4) | 3 (7) |  |
| Stable disease | 6 (9) | 519) | 1 (2) |  |
| Progression of disease | 8 (11) | 4 (15) | 4 (10) |  |

Abbreviations: CR- complete response, VGPR-very good partial response, CL- circulating lymphoma

**Table S2. Univariate and Multivariate Cox modeling on time to treatment among patients who received systemic therapy**

| **Variable** | **Univariate** | | **Multivariate** | |
| --- | --- | --- | --- | --- |
|  | **HR (95% CI)** | **P-value** | **HR (95% CI)** | **P-value** |
| Circulating Lymphoma |  |  |  |  |
| CL- | 1.00 |  |  |  |
| CL+ | 1.64 (1.00 – 2.69) | **0.05** | 1.43 (0.78 – 2.61) | 0.25 |
| Age at dx (yrs) | 0.99 (0.97 – 1.01) | 0.42 | - | - |
| Sex |  |  |  |  |
| Male | 1.00 |  | - | - |
| Female | 1.05 (0.64 – 1.71) | 0.86 | - | - |
| Race |  |  |  |  |
| White | 1.00 |  | - | - |
| Black | 0.58 (0.30 – 1.15) | 0.12 | - | - |
| ECOG PS |  |  |  |  |
| 0-1 | 1.00 |  | - | - |
| ≥ 2 | 2.17 (0.85 – 5.57) | 0.11 | - | - |
| IgM level |  |  |  |  |
| ≤ 1000 | 1.00 |  |  |  |
| 1001 – 3000 | 0.80 (0.43 – 1.48) | 0.48 | 0.88 (0.43 – 1.82) | 0.74 |
| > 3000 | 1.91 (1.04 – 3.52) | **0.04** | 2.38 (1.21 – 4.71) | **0.01** |
| B Symptoms |  |  |  |  |
| No | 1.00 |  |  |  |
| Yes | 3.69 (1.55 – 8.74) | **0.003** | 3.59 (1.28 – 10.09) | **0.02** |
| Albumin Low |  |  |  |  |
| No | 1.00 |  |  |  |
| Yes | 2.10 (1.21 – 3.64) | **0.01** | 1.20 (0.54 – 2.65) | 0.66 |
| LDH > ULN |  |  |  |  |
| No | 1.00 |  |  |  |
| Yes | 1.61 (0.93 – 2.79) | 0.09 | 1.43 (0.71 – 2.90) | 0.32 |
| Elevated B2M |  |  |  |  |
| No | 1.00 |  | - | - |
| Yes | 1.73 (0.85 – 3.51) | 0.13 | - | - |
| Complex Karyotype |  |  |  |  |
| No | 1.00 |  |  |  |
| Yes | 1.79 (1.07 – 3.02) | **0.03** | 1.05 (0.53 – 2.05) | 0.90 |
| Prognostic score (IPSSWM) |  |  |  |  |
| 0-1 | 1.00 |  |  |  |
| 2 | 1.95 (1.09 – 3.50) | **0.03** | 1.70 (0.82 – 3.52) | 0.15 |
| 3-4 | 2.26 (1.21 – 4.22) | **0.01** | 2.43 (1.08 – 5.49) | **0.03** |

Abbreviations: Yrs- years, CL- circulating lymphoma, ECOG PS- Eastern Cooperative Oncology Group performance status, LDH- lactate dehydrogenase, ULN- upper limit of normal, B2M- beta-2-microglobulin, IPSSWM- International Prognostic Scoring System for Waldenstrom Macroglobulinemia

**Table S3. Studies that reported the incidence of circulating lymphoma cells in WM.**

| **Study** | **Sample size (N)** | **Prevalence of CL**  **N (%)** | **Key Findings** |
| --- | --- | --- | --- |
| Pettersson et al., *Scand* *J Immunol*, 1980. | 16 | 9 (56%) | Larger fraction of PB monoclonal population was associated with advanced disease (splenomegaly, lymph node enlargement). |
| Smith et al., *Blood*, 1983. | 12 | 12 (100%) | Absolute number of circulating abnormal lymphocytes was not correlated with IgM level or symptoms at diagnosis.  Clinical response to therapy was associated with corresponding decreased in abnormal PB cells after treatment. |
| Oertel et al., *Clin* *Lab* *Haematol*, 2002 | 5 | 3 (60%) | 3-17% range of lymphoplasmacytoid cells as percentage of PB leukocytes (n=3) |
